# Where Morphological and Molecular Classifications Meet: The Role of p53 Immunohistochemistry in the Prognosis of Low-Risk Endometrial Carcinoma (GLAMOUR Study)

**DOI:** 10.3390/cancers16061088

**Published:** 2024-03-07

**Authors:** Andrea Puppo, Giulio Fraternali Orcioni, Valentino Clignon, Yuri Musizzano, Carla Angela Zavattero, Giulia Vocino Trucco, Giacomo Maria Benazzo, Giuseppe Vizzielli, Stefano Restaino, Laura Mariuzzi, Maria Orsaria, Renato Seracchioli, Diego Raimondo, Linda Bertoldo, Stefano Uccella, Anna Caliò, Giulia Vittori Antisari, Simone Garzon, Vito Andrea Capozzi, Roberto Berretta, Francesco Cosentino, Alfredo Ercoli, Antonio Ieni, Martina Arcieri, Marcello Ceccaroni, Anna Pesci, Giulia Mantovani, Francesco Bruni, Giovanni Roviglione, Pio Zeppa, Antonio Raffone, Marco Camanni, Elena Maria Delpiano, Claudia Provenza, Martina Borghese, Giuseppe Migliaretti

**Affiliations:** 1 Department of Gynecology and Obstetrics, Azienda Sanitaria Ospedaliera Santa Croce e Carle, 12100 Cuneo, Italy; puppo.a@ospedale.cuneo.it (A.P.); clignon.v@ospedale.cuneo.it (V.C.); 2 Department of Pathology, Azienda Sanitaria Ospedaliera Santa Croce e Carle, 12100 Cuneo, Italy; fraternali.g@ospedale.cuneo.it (G.F.O.); y.musizzano@asl2.liguria.it (Y.M.); 3 Department of Pathology, Ospedale Regina Montis Regalis, 12084 Mondovì, Italy; carlaangela.zavattero@aslcn1.it (C.A.Z.); giacomo.benazzo@edu.unito.it (G.M.B.); 4 Clinic of Obstetrics and Gynecology, “Santa Maria della Misericordia” University Hospital, Azienda Sanitaria Universitaria Friuli Centrale, 33100 Udine, Italy; giuseppevizzielli@yahoo.it; 5 Department of Medicine, University of Udine, 33100 Udine, Italy; 6 Clinic of Obstetrics and Gynecology, “Santa Maria della Misericordia” University Hospital, Azienda Ospedaliera Sanitaria Universitaria Friuli Centrale, 33100 Udine, Italy; stefano.restaino@asufc.sanita.fvg.it; 7 Institute of Pathology, Academic Hospital “Azienda Sanitaria Universitaria Integrata di Udine”, 33100 Udine, Italy; laura.mariuzzi@uniud.it (L.M.); maria.orsaria@asufc.sanita.fvg.it (M.O.); 8 Institute of Pathology, DAME (Medical Area Department), University of Udine, 33100 Udine, Italy; 9 Division of Gynecology and Human Reproduction Physiopathology, IRCCS Azienda Ospedaliero-Universitaria di Bologna, 40138 Bologna, Italy; renato.seracchioli@unibo.it (R.S.); diego.raimondo@aosp.bo.it (D.R.); linda.bertoldo@studio.unibo.it (L.B.); 10 Department of Obstetrics and Gynecology, AOUI Verona, University of Verona, 37129 Verona, Italy; stefano.uccella@univr.it (S.U.); giulia.vittori@sacrocuore.it (G.V.A.); simone.garzon@univr.it (S.G.); 11 Department of Diagnostic and Public Health, Section of Pathology, University of Verona, 37129 Verona, Italy; anna.calio@univr.it; 12 Department of Medicine and Surgery, University Hospital of Parma, 43126 Parma, Italy; vitoandrea.capozzi@studenti.unipr.it (V.A.C.); roberto.berretta@unipr.it (R.B.); 13 Department of Medicine and Science of Life “Vincenzo Tibero”, University of Molise UNIMOL, 86100 Campobasso, Italy; francesco.cosentino@gemellimolise.it; 14 Department of Human Pathology in Adult and Developmental Age “Gaetano Barresi”, University of Messina, 98125 Messina, Italy; aercoli@unime.it (A.E.); antonio.ieni@unime.it (A.I.); 15 Department of Biomedical Dental, Morphological and Functional Imaging Science, University of Messina, 98125 Messina, Italy; martina.arcieri@unime.it; 16 Department of Obstetrics and Gynecology, Gynecologic Oncology and Minimally-Invasive Pelvic Surgery, International School of Surgical Anatomy, IRCCS “Sacro Cuore—Don Calabria” Hospital, 37024 Negrar di Valpolicella, Italy; mceccaroni@libero.it (M.C.); giulia.mantovani@sacrocuore.it (G.M.); francesco.bruni@sacrocuore.it (F.B.); giovanni.roviglione@sacrocuore.it (G.R.); 17 Department of Pathology, IRCCS “Sacro Cuore—Don Calabria” Hospital, 37024 Negrar di Valpolicella, Italy; anna.pesci@sacrocuore.it; 18 Pathology Department, University Hospital San Giovanni e Ruggi d’Aragona, 84131 Salerno, Italy; pzeppa@unisa.it; 19 Gynecology and Obstetrics Unit, Department of Neuroscience, Reproductive Sciences and Dentistry, School of Medicine, University of Naples Federico II, 80128 Naples, Italy; antonio.raffone2@unibo.it; 20 Department of Medical and Surgical Sciences (DIMEC), University of Bologna, 40138 Bologna, Italy; 21 Department of Gynecology and Obstetrics, ASL Citta’ di Torino, 10126 Torino, Italy; marco.camanni@aslcittaditorino.it (M.C.); delpi_eli@yahoo.com (E.M.D.); 22 Department of Pathology, ASL Citta’ di Torino, Ospedale Giovanni Bosco, 10126 Torino, Italy; claudia.provenza@aslcittaditorino.it; 23 Department of Public Health and Pediatric Sciences, University of Turin, 10126 Turin, Italy; giuseppe.migliaretti@unito.it

**Keywords:** endometrial cancer, molecular classification, p53, target therapy

## Abstract

**Simple Summary:**

There is a lack of literature on the role of molecular classification in patients with morphological low-risk EC. We aimed to evaluate the incidence and prognostic role of p53 mutations in this specific subgroup of patients. Our findings show that 4.9% of low-risk EC are p53abn; the OR for the recurrence of p53abn versus p53wt patients was 5.23—CI 95% 0.98–27.95, *p* = 0.053. No difference in OS was observed between the two groups. Recurrences were mostly local and occur two years after diagnosis. Our data might serve as a valuable tool for clinicians’ everyday practice, but larger prospective studies are urgently needed.

**Abstract:**

No prospective study has validated molecular classification to guide adjuvant treatment in endometrial cancer (EC), and not even retrospective data are present for patients with morphological low-risk EC. We conducted a retrospective, multicenter, observational study including 370 patients with low-risk endometrioid EC to evaluate the incidence and prognostic role of p53 abnormal expression (p53abn) in this specific subgroup. Among 370 patients, 18 had abnormal expressions of p53 (4.9%). In 13 out of 370 patients (3.6%), recurrences were observed and two were p53abn. When adjusting for median follow-up time, the odds ratio (OR) for recurrence among those with p53abn versus p53 wild type (p53wt) was 5.23—CI 95% 0.98–27.95, *p* = 0.053. The most common site of recurrence was the vaginal cuff (46.2%). One recurrence occurred within the first year of follow-up, and the patient exhibited p53abn. Both 1-year and 2-year DFS rates were 94.4% and 100% in the p53abn and p53wt groups, respectively. One patient died from the disease and comprised p53wt. No difference in OS was registered between the two groups; the median OS was 21.9 months (16.4–30.1). Larger multicenter studies are needed to tailor the treatment of low-risk EC patients with p53abn. Performing molecular classification on all EC patients might be cost-effective, and despite the limits of our relatively small sample, p53abn patients seem to be at greater risk of recurrence, especially locally and after two years since diagnosis.

## 1. Introduction

Endometrial cancer (EC) is the most common gynecological malignancy in industrialized countries with a steadily increasing trend [1]. Although prognosis in early stages is mostly favorable with a 5-year overall survival of about 95% [2,3], around 20% of patients with early-stage disease have an unfavorable prognosis [4,5,6].

The new molecular classification introduced by the Cancer Genome Atlas (TGCA) defined four molecular risk groups based on mutational burden: ultramutated tumors with polymerase epsilon (POLE) mutations that have excellent prognoses, microsatellite instability (MSI), a low copy number (known as NSMP) with intermediate prognosis, and a high copy number with frequent tumor protein p53 (TP53) alterations and poor prognoses. This classification was added to the older morphological evaluation with major consequences on EC prognosis, diagnosis, and treatment [7,8,9,10].

Patients with TP53-mutated tumors, accounting for about 15% of all EC diagnoses and 10% of morphological low-risk cases, have the worst prognosis with up to 50–70% of all EC mortality [8,11]. According to the latest guidelines, this subgroup of high-risk patients benefits from adjuvant treatment with chemotherapy or sequential/concurrent radiotherapy [3].

To date, no prospective study has validated the use of molecular classification to guide adjuvant treatment in EC, and to our knowledge, not even retrospective data are present for patients with morphological low-risk EC [12,13]. Notably, the ongoing prospective randomized PORTEC-4 [14] and RAINBO [15] studies, designed with molecular-integrated risk profile-based recommendations, will introduce exceptional results that will shape the future management of EC, but the first one still does not include patients with very low-risk EC.

The main rationale of our study is to evaluate the incidence and prognostic role of molecular profiles, particularly the role of abnormal p53 immunohistochemical expression (p53abn) in patients with morphological low-risk EC.

## 2. Materials and Methods

### 2.1. Study Design

A retrospective, multicenter, observational study was conducted, including 370 patients with low-risk endometrioid EC treated from 1 January 2016 to 31 December 2020 in 10 different Italian gynecologic oncology departments (Santa Croce e Carle in Cuneo, Santa Maria della Misericordia in Udine, Policlinico Sant’Orsola in Bologna, Azienda Ospedaliera Universitaria Integrata di Verona, Ospedale Maggiore in Parma, Fondazione Giovanni Paolo II in Campobasso, Policlinico Martino in Messina, Sacro Cuore Don Calabria in Negrar, Ospedale di Polla S. Arsenio in Salerno, and Ospedale Martini in Torino).

Trained medical doctors reviewed operative room registers and gynecologic oncology databases to identify all low-risk endometrioid EC patients who underwent surgery and a subsequent follow-up of at least 24 months at each study center. Low-risk endometrioid EC was defined as FIGO stage IA, G1-G2 endometrioid, and lymph-vascular space invasion (LVSI) negative EC. The final pathology report of primary surgery was used for case classification, based on FIGO staging according to the 2009 revised classification system. POLE mutation testing was included only in a minority of cases due to the recent introduction of the method [3].

**Figure 1:**
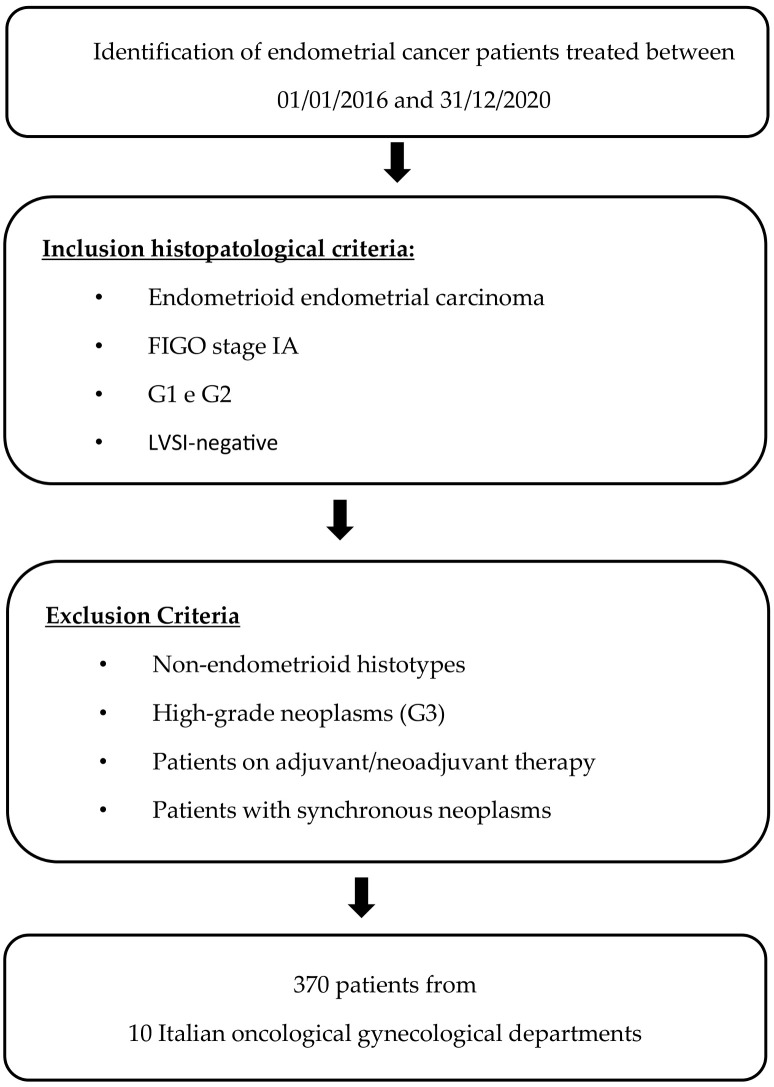
Development process.

Among identified patients with low-risk endometrioid EC, those who underwent neoadjuvant or adjuvant treatment or had synchronous malignancy were excluded. Of the included patients, clinicopathological, surgical, and survival data were extracted from medical records. When follow-up information was updated in December 2022, telephone contact was made with the patients or their relatives. All patients attended follow-up visits at all study centers according to ESGO guidelines [3] (Figure 1).

As a primary outcome, we evaluated the incidence of p53abn (immunohistochemical surrogate for TP53 molecular alterations). p53 immunohistochemistry patterns were defined as follows: (1) wild-type pattern (normal p53 IHC): when the distribution of nuclear staining in a “wild type” pattern ranges from a few positive cells to almost all cells being stained, but with variable intensity; (2) hyperexpression (abnormal p53 IHC): defined as strong nuclear staining in at least 80% of tumor cell nuclei; (3) complete absence or null pattern (abnormal): defined as no staining in tumor cell nuclei in the presence of the “wild-type”; (4) cytoplasmic (abnormal): defined as predominant cytoplasmic staining in the absence of strong nuclear staining in >80% of tumor cell nuclei, (5) subclonal expression (abnormal): defined as the combination of normal with one or more abnormal patterns;(6) inconclusive: when none of the last criteria are met [16,17,18].

All centers that participated in the study utilized the same analysis method for the study of p53.

As a secondary endpoint, we evaluated patients’ survival in terms of disease-free survival (DFS), defined as the time from the date of diagnosis to the detection of recurrence or the latest observation, and overall survival (OS), defined as the time from the date of primary surgery to death or the latest observation. Recurrence was defined as the histological evidence of the original disease after primary surgical treatment. We also analyzed the most common histopathological and clinical characteristics associated with the abnormal expression of p53.

All research activities were approved by the Institutional Review Board (16 March 2022—N° PROT. APROV. 51-2022), and written informed consent was waived by the IRB because patients’ data were collected anonymously.

### 2.2. Statistical Analysis

Descriptive statistical indicators were estimated to describe clinical and demographic characteristics. Quantitative variables will be summarized with the mean, standard deviation, min, and max. Qualitative variables will be described using frequency (absolute and percentage) tables. The incidence of the p53abn protein in patients with FIGO stage IA G1-G2, LVSI-negative EC, and response and recurrence rates were estimated and shown with relative 95%. confidence intervals. PFS and OS were analyzed using Kaplan–Meier curves and a relative 95% confidence interval. All statistical analyses were performed using SAS^®^ and STATA Statistics Software (STATA 18 Version, year 2023) [19,20,21].

## 3. Results

### 3.1. General Demographic and Clinical Data

Between 1 January 2016 and 31 December 2020, 370 consecutive low-risk endometrioid EC patients underwent surgery at ten Italian centers. The median follow-up was 34.2 months (range of 32.3–36.1).

The average age of patients in the study population was 63. Most patients were postmenopausal (89.7%) and overweight (body mass index > 30 kg/m^2^) (31.9%). The family history of EC was reported by 2.9% of women. Vaginal bleeding was the main symptom at onset (74.1% of cases), while five patients (1.4%) presented with abnormal pap smear results. Fourteen patients (3.8%) had a positive remote pathological history for gynecological cancers, and preoperatively, 69.2% of women were correctly diagnosed with stage FIGO IA via ultrasound. In total, 162 patients (43.8%) underwent peritoneal washing cytology (PWC), radical hysterectomy (RH), and bilateral salpingo-oophorectomy (BSO), while all others underwent concomitant retroperitoneal staging, either by sentinel lymph node (SLN) evaluation and/or radical lymphadenectomy (Table 1).

### 3.2. Histopathological and Immunohistochemical Characteristics

Histopathological and immunohistochemical characteristics of the 370 patients are reported in Table 2.

Among p53abn patients, 16 (88.9%) had G1 tumors versus 201 (57.1%) in the p53wt group (*p*-value *p* = 0.007). The MELF pattern was present in 1 patient in the p53abn group (5.6%) versus 39 (11.1%) in the p53wt group (Table 3).

In both groups, the majority of women were p-MMR (15 (83.3%) for MSH-6 and MSH-2 and 18 (100%) for PMS-2 and MLH-1 in p53abn patients and 333 (94.6%) for MSH-6, 334 (97.7%) for MSH-2, 294 (83.5%) for PMS-2, and 297 (84.5%) for MLH-1 in p53wt patients).

The vast majority of patients in both groups had tumors expressing hormonal receptors (18 (94.4%) for both ER and PgR in p53abn patients and 343 (97.4%) and 335 (95.2%) for ER and PgR, respectively, in the p53wt group) (Table 3). A statistically significant difference was found between the two groups concerning MSH-2 expression (*p* = 0.006) and POLE mutation (*p* = 0.007).

In total, 216 patients (58.4%) had G1 tumors and 154 (41.6%) had G2. Microcystic elongated and fragmented (MELF) patterns were present in 10.5% of women. Regarding mismatched repair proteins, most patients were proficient (p-MMR): 93.5% for MSH-6, 93.2% for MSH-1, 70.3% for PMS-2, and 81.6% for MLH-1. In the majority of patients, ER was expressed in more than 50% of cells (72.7%), and PgR was also expressed in 56.8%. POLE analysis was carried out in 11.6% of cases, and no patient carried this mutation.

Most patients (95.1%) exhibited the normal IHC expression of p53 (wild-type—wt), while 18 patients exhibited abnormal expression (4.9%). Among these, seven had abn hyp expression (1.9%), eight had abn null (2.2%), two had abn cyt (0.5%), and one had abn sub (0.3%).

The average age of p53abn women was 65 versus 63 in p53wt patients. p53-mutated patients were postmenopausal in 100% of cases versus 86.4% in the p53wt group, and overweight was observed in 55.6% versus 79.5% in the p53wt group. In both groups, most patients underwent PWC, RH, BSO, and SLN biopsies (61.1% and 36.1% for p53abn and p53wt patients, respectively). Among clinical data, the only statistically significant data that differed between the two study groups were those about oncological family history (*p* = 0.0001) and the type of surgery (*p* = 0.0002).

### 3.3. Survival Analysis

Survival data are provided in Table 4.

Four patients were lost at follow-up. The mean follow-up time from initial diagnosis was 34.2 months (32.3–36.1). The median follow-up of patients with p53abn was 22.2 months (IC 95% 15.4–29.0) versus 34.9 months (IC 95% 32.95–36.79) with respect to patients with p53wt.

At the last follow-up (December 2022), 359 (98.1%) patients were alive without evidence of disease, 3 (0.8%) were alive with disease, 1 (0.3%) died due to disease, and 3 patients (0.8%) died from causes other than EC that had never previously recurred.

There were 13 recurrences among our cohort (3.6%). Among patients who recurred, two patients were p53abn, and the OR for recurrence for patients with p53abn was 3.83 (95% CI 0.78–18.74) (*p* = 0.09). When adjusting for median follow-up time, the OR for recurrence among those with p53abn versus p53 wild type (p53wt) was 5.23—CI 95% 0.98–27.95—which was at the limits and exhibited statistical significance (*p* = 0.053).

Only one recurrence was registered within the first year of follow-up; this patient was p53abn.

**Figure 2:**
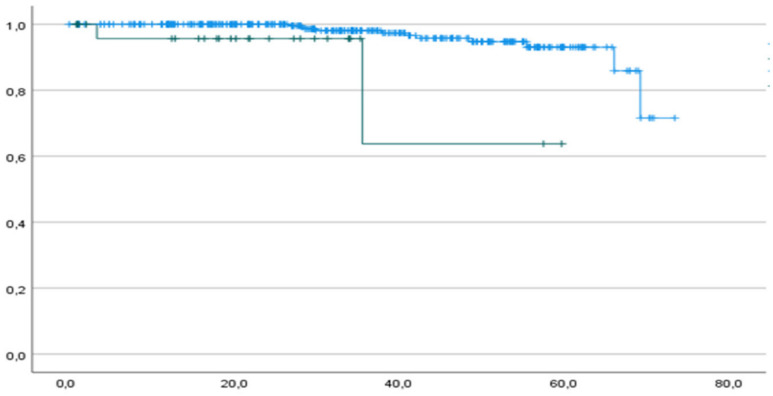
DFS of p53abn patients (green curve) and p53wt patients (blue curve). Horizontal axis (*x*-axis): months since surgery. Vertical axis (*y*-axis): cumulative survival.

Both 1-year and 2-year DFS rates were 94.4% and 100% in the p53abn and p53wt groups, respectively, and the median DFS was 21.8 (IC 95% 15.4–29.0) and 34.2 months (IC 95% 32.6–36.6) for mutated and wt patients, respectively (Figure 2).

In Table 5, we reported the main characteristics of the two p53abn patients who recurred.

The most common site of recurrence was the vaginal cuff (six patients, 46.1%), followed by isolated pelvic peritoneal and isolated parenchymatous (two patients and 15.4% each). Eight patients (61.5%) underwent surgery, four (30.8%) received radiotherapy only, and only one patient (7.7%) was treated with chemotherapy at relapse

**Figure 3:**
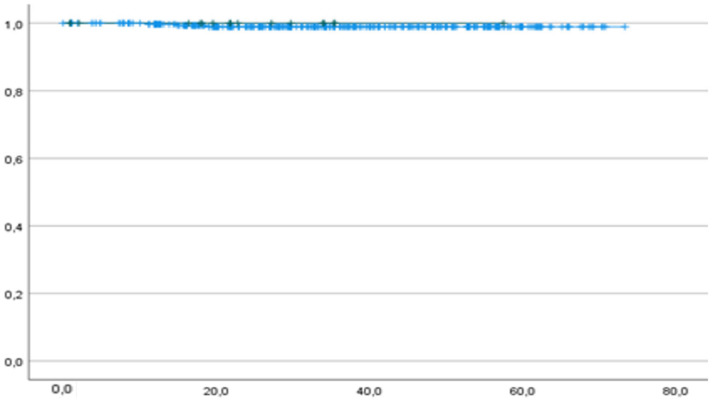
OS of p53abn (green curve) and p53wt (blue curve) patients. *p* = 0.709. Horizontal axis (*x*-axis): months since surgery. Vertical axis (*y*-axis): cumulative survival.

Only one patient died from the disease (0.3%), and she had p53wt. One-year OS was 100% and 99.7% in the p53abn and p53wt groups, respectively. Two-year OS was 100% and 99.1% in the p53abn and p53wt groups, respectively. The median OS was 21.9 months (IC 95% 16.4–30.1) for p53abn and 34.2 months (IC 95% 33.2–37.0) for p53wt patients (Figure 3).

## 4. Discussion

The study describes the prevalence of p53abn in a cohort of low-risk EC patients (4.9%) and aims to assess whether the p53 mutation is associated with survival outcomes in low-risk patients to help clinicians manage their patients in everyday clinical practice. The plausible poor prognostic significance of the p53 mutation was confirmed by the recent 2023 FIGO staging guidelines that upstaged p53abn FIGO IA (2009) tumors relative to FIGO stage IIC [22]. None of the studies from which such guidelines were formulated were included; however, very low-risk morphological patients and the major ongoing prospective studies investigating molecular profiles have not included such subgroups of low-risk patients either [14].

A reliable strategy to identify patients at high risk of recurrence despite low morphological risk is urgently needed. There are plenty of data in the literature regarding the prognostic role of molecular classifications on EC [9,23]; less clear is its role in guiding adjuvant treatment since prospective studies are missing to date.

Molecular profiling is recommended routinely in all patients with EC [3]. According to the current guidelines, POLE mutation should be performed as the first analysis [24]; however, as most patients are diagnosed with low morphological risk, it is questionable whether such an approach is cost-effective in clinical practice, especially since POLE analysis cannot routinely be performed for the paucity of laboratories and high costs [25]. We also need to consider the real use of molecular data in daily clinical practice in this particular subgroup of women in the absence of literature data that allows clinicians to tailor adjuvant treatments.

In a recent study by Vrede et al. [26], tumor molecular profiles were not associated with the outcomes in patients with low-grade EC, and the authors concluded that molecular classification could be omitted for this subgroup.

In our opinion, POLE analysis should be performed if not previously carried out in cases of low-risk p53abn EC to exclude multiple classifiers [27].

According to a recent study, the characteristics of POLEmut-p53abn resembled those of POLEmut, characterized by an excellent prognosis in the absence of adjuvant therapies [28].

We also strongly suggest analyzing p53 mutations in such settings using next-generation sequencing (NGS) and not only immunohistochemistry since this technique is more reliable in reducing the rate of false positives [29].

In our cohort, 2 out of 13 patients who recurred had p53abn (15%): Despite the limitations of a small sample, p53abn patients seem to be at a greater risk of recurrence, especially locally and two years after diagnosis. When adjusting for the median follow-up time, recurrence risk was higher for p53abn patients (OR 5.23–CI 95% 0.98–27.95; *p* = 0.053). The results remain consistent even at the limits of statistical significance, and this is probably due to an overestimation of the beta error caused by the low incidence of p53abn in the study group. The sample size of the two groups is actually very different, and the higher risk for the recurrence of p53abn should be considered in light of these data.

A worse DFS starts mainly after 40 months among our patients, but the curves already differ within the first 35 months (Figure 2). We registered one recurrence within the first year of follow-up, and this patient had p53abn; the remaining 12 recurrences occurred after 24 months, and only 1 had p53abn. Among patients who recurred, two had p53abn (Table 5).

In total, four patients within our cohort died (1.1%), and out of these, one (0.3%) was a result of EC. In particular, this 64-year-old patient comprised the p53 wild type, and her tumor was p-MMR; ER was positive in 60% of cells and was PgR (in 40%). She recurred in the vaginal cuff and pelvic lymph nodes after six months, and she finally died of the disease (OS 28 months).

There was no significant difference between the p53abn and p53wt groups regarding OS (Figure 3).

Our study is strengthened by being the only study in the literature that has so far investigated patients with p53abn and low morphological risks. Moreover, apart from giving a detailed description of the prevalence of such mutations in low-risk patients, we also concentrated on possible clinical, histopathological, and survival associations to understand the physiopathology of this disease and pave the way for tailored treatment more substantially.

The findings of this study must be observed while considering some limitations: The number of events was too low in the subgroups of analysis to extrapolate solid conclusions. Some ways to overcome this limitation may be conducting larger multicenter studies that include bigger cohorts comprising low-risk EC patients. Moreover, our study embraces a relatively long timeframe, which was necessary for recruiting more cases, but this resulted in more heterogeneity in the treatment, especially since the sentinel node algorithm [3] and POLE analysis were introduced. In accordance with ESGO guidelines, not all patients with very low-risk morphological endometrial cancer underwent retroperitoneal staging, and this could have upgraded the tumor stage [3,30]. Lastly, the median follow-up time differed significantly for the two groups (22.2 versus 34.9 months in p53abn versus p53wt patients).

## 5. Conclusions

Given the rarity of p53abn EC in morphological low-risk EC, the literature is extremely limited. Since no data from prospective studies allow us to modify the adjuvant therapy of low-risk women based on molecular analysis so far, we at least suggest intensifying the follow-up of p53abn women. Based on our study, although within the described limits, we recommend that clinicians pay attention primarily to the local recurrence of p53abn tumors, even after two years since the primary treatment.

We believe that the incidence of p53abn in morphological low-risk EC patients (4.9%) is sufficiently high to make it advisable to investigate p53 protein statuses in all EC patients, particularly since the diagnosis of p53abn is possible in routine clinical practice with relatively low costs and high reproducibility. This analysis is even feasible preoperatively on endometrial biopsy specimens, and this may be particularly relevant for young patients who desire to preserve their fertility [31]. As mentioned, we need to keep in mind that the p53 status only has a prognostic role so far, and it is not yet sufficiently strong for modifying adjuvant treatment in low-risk EC patients.

## Figures and Tables

**Table 1:** Demographic and clinical characteristics of 370 women.

Characteristics	Number of Cases (%)
**All cases**	370 (100%)
**Age (years)**	
Average (range)	63 (32–86)
**Parity**	
Previous pregnancies	283 (76.5%)
Nulliparous	87 (23.5%)
**Menopause**	
Yes	332 (89.7%)
No	38 (10.3%)
**Body Mass Index**	
<18.5	3 (0.7%)
18.5–24.9	76 (20.5%)
25–30	118 (30.9%)
30.1–34.9	63 (18.5%)
35–40	35 (9.3%)
>40	38 (10.1%)
Unknown	37 (10%)
**Family history of gynecological cancer**	
No	278 (75.3%)
Yes (endometrial)	11 (2.9%)
Yes (ovary)	11 (2.9%)
Yes (breast)	19 (5.3%)
Unknown	51 (13.6%)
**Other previous tumors**	
None	325 (87.8%)
Yes (gynecologic)	14 (3.8%)
Yes (breast)	9 (2.5%)
Yes (bowel)	12 (3.2%)
Other	10 (2.7%)
**Symptoms at diagnosis**	
Vaginal bleeding	309 (83.5%)
Abdominal pain/swelling	15 (4.1%)
Vaginal bleeding and abdominal pain/swelling	11 (2.9%)
Other	35 (9.5%)
**Preoperative histological diagnosis**	
Endometrioid G1-2	302 (81.6%)
Endometrial hyperplasia	34 (9.2%)
Other histology	17 (4.6%)
Not carried out	17 (4.6%)
**Pap smear**	
Normal	194 (52.4%)
Inflammatory	8 (2.2%)
Malignancy	5 (1.4%)
Not a recent analysis (>18 months)	163 (44%)
Preoperative radiologic FIGO staging *	
Stage Ia	265 (71.6%)
Stage Ib	44 (11.9%)
Stage II	4 (1.1%)
Stage III	0 (0%)
Stage IV	1 (0.3%) **
Unknown	56 (15.1%)
**Type of surgery**	
PWC, RH + BSO + SLN	141 (38.1%)
PWC, RH + BSO + P lymph nodes	49 (13.2%)
PWC, RH + BSO + P and PA lymph nodes	5 (1.4%)
PWC, RH + BSO + SLN + P and/or PA lymph nodes	13 (3.5%)
PWC, RH + BSO	162 (43.8%)

PWC = peritoneal washing cytology. RH = radical hysterectomy. BSO = bilateral salpingo-oophorectomy. SLN = sentinel lymph node biopsy. P = pelvic. PA = para-aortic. * = either with ultrasound or CT scan. ** = suspect of IV stage at preoperative diagnosis, not confirmed by intraoperative finding or histology.

**Table 2:** Histopathological and immunohistochemical characteristics of 370 women. WT = “wild type” expression; Abn hyp = abnormal hyperexpression; Abn null = abnormal “null” expression; Abn cyt = abnormal cytoplasmic expression; Abn sub = abnormal subclonal; ER = estrogen; PgR = progesterone.

**Grading**	
G1	216 (58.4%)
G2	154 (41.6%)
**MELF pattern**	
Absent	294 (79.5%)
Present	39 (10.5%)
Unknown	37 (10%)
**p53 status**	
WT	352 (95.1%)
Abn hyp	7 (1.9%)
Abn null	8 (2.2%)
Abn cyt	2 (0.5%)
Abn sub	1 (0.3%)
**MSH-6**	
Preserved	346 (93.5%)
Lost	21 (5.7%)
Unknown	3 (0.8%)
**MSH-2**	
Preserved	345 (93.2%)
Lost	10 (2.7%)
Unknown	15 (4.1)
**PMS-2**	
Preserved	296 (80.0%)
Lost	60 (16.2%)
Unknown	14 (3.8%)
**MLH-1**	
Preserved	302 (81.6%)
Lost	55 (14.9%)
Unknown	13 (3.5%)
**ER (%)**	
Negative	9 (2.4%)
Positive <25%	30 (8.1%)
Positive 25–49%	62 (16.8%)
Positive ≥50%	269 (72.7%)
**PgR (%)**	
Negative	15 (4.1%)
Positive <25%	59 (15.9%)
Positive 25–49%	86 (23.2%)
Positive ≥50%	210 (56.8%)
**POLE**	
Not carried out	327 (88.4%)
Mutated	0 (0%)
Nonmutated	43 (11.6%)

**Table 3:** Comparison between the characteristics of the 18 p53abn patients and the 352 p53wt patients.

Characteristics	p53abn	p53wt	*p* Value *
	Patients (%)	Patients (%)	
**All cases**	18 (100%)	352 (100%)	
**Age (years)**			
Average (range)	65 (49–81)	63 (32–86)	*p* = 0.721
**Parity**			
Previous pregnancies	18 (100%)	272 (77.3%)	
Nulliparous	0 (0%)	80 (22.7%)	*p* = 0.078
**Menopause**			
Yes	18 (100%)	304 (86.4%)	
No	0 (0%)	48 (13.6%)	*p* = 0.294
**Body Mass Index**			
Underweight	0 (0%)	3 (0.9%)	
Normal weight	7 (38.9%)	69 (19.6%)	
Overweight	10 (55.6%)	244 (69.3%)	
Unknown	1 (5.5%)	36 (10.2%)	*p* = 0.071
**Family history of gynecological cancer**			
EC	7 (38.9%)	2 (0.6%)	
OC	0 (0%)	11 (3.1%)	
BC	0 (0%)	19 (5.4%)	
No	11 (61.1%)	262 (74.4%)	
Unknown	0 (0%)	58 (16.5%)	*p* = 0.0001
**Symptoms at diagnosis**			
Vaginal bleeding	16 (88.8%)	304 (86.4%)	
Abdominal pain/swelling	0 (0%)	15 (4.2%)	
Others	2 (11.2%)	33 (9.4%)	*p* = 0.962
**Preoperative histological diagnosis**			
Endometrioid G1-2	16 (88.8%)	295 (83.8%)	
Endometrial hyperplasia	2 (11.2%)	32 (9.1%)	
Other histology	0 (0%)	8 (2.3%)	
Not carried out	0 (0%)	17 (4.8%)	*p* = 0.881
**Pap smear**			
Normal	11 (61.1%)	183 (51.9%)	
Inflammatory	0 (0%)	8 (2.3%)	
Malignancy	0 (0%)	5 (1.4%)	
Not a recent analysis (>18 months)	7 (38.9%)	156 (44.4%)	*p* = 0.476
Preoperative radiologic FIGO staging **			
Stage Ia	16 (88.8%)	249 (70.7%)	
Stage Ib	2 (11.2%)	42 (11.9%)	
Stage ≥II	0 (0%)	5 (1.4%)	
Unknown	0 (0%)	56 (16.0%)	*p* = 0.353
**Type of surgery**			
PWC, RH + BSO + SLN	11 (61.1%)	127 (36.1%)	
PWC, RH + BSO + P lymph	2 (11.1%)	47 (13.4%)	
PWC, RH + BSO + P and PA lymph nodes	0 (0%)	5 (1.4%)	
PWC, RH + BSO + SLN + P and/or PA lymph	4 (22.2%)	10 (2.8%)	
PWC, RH + BSO	1 (5.6%)	163 (46.3%)	*p* = 0.0002
**Grading**			
G1	16 (88.9%)	201 (57.1%)	
G2	2 (11.1%)	151 (42.9%)	*p* = 0.007
**MELF pattern**			
Absent	17 (94.4%)	290 (82.4%)	
Present	1 (5.6%)	39 (11.1%)	
Unknown	0 (0%)	23 (6.5%)	*p* = 0.697
**MSH-6**			
Preserved	15 (83.3%)	333 (94.6%)	
Lost	3 (16.7%)	19 (5.4%)	*p* = 0.051
**MSH-2**			
Preserved	15 (83.3%)	334 (97.7%)	*p* = 0.006
Lost	3 (16.7%)	8 (2.3%)	
**PMS-2**			
Preserved	18 (100%)	294 (83.5%)	
Lost	0 (0%)	58 (16.5%)	*p* = 0.193
**MLH-1**			
Preserved	18 (100%)	297 (84.5%)	
Lost	0 (0%)	55 (15.5%)	*p* = 0.231
**ER (%)**			
Negative	1 (5.6%)	9 (2.6%)	
Positive <25%	1 (5.6%)	37 (10.5%)	
Positive 25–49%	0 (0%)	36 (10.2%)	
Positive ≥50%	16 (88.8%)	270 (76.7%)	*p* = 0.671
**PgR (%)**			
Negative	1 (5.6%)	17 (4.8%)	
Positive <25%	1 (5.6%)	34 (9.6%)	
Positive 25–49%	0 (0%)	65 (18.5%)	
Positive ≥50%	16 (88.8%)	236 (67%)	*p* = 0.401
**POLE**			
Not carried out	13 (72.2%)	327 (92.9%)	
Mutated	0 (0%)	0 (0%)	
Nonmutated	5 (27.8%)	25 (7.1%)	*p* = 0.007

OC = ovarian cancer; BC = bowel cancer; PWC = peritoneal washing cytology. RH = radical hysterectomy. BSO = bilateral salpingo-oophorectomy. SLN = sentinel lymph node biopsy. P = pelvic. PA = para-aortic; ER = estrogen; PgR = progesterone. * = either with ultrasound or CT scan. ** = results are presented without the necessary adjustments for multiple comparisons that would downsize the effect due to the mere descriptive nature of the table and considering that the only data that significatively differ do not influence the final data of the study.

**Table 4:** Follow-up characteristics and survival data of 370 women.

Characteristics	Number of Cases (%)
**Follow up**	
Patients lost at follow-up	4 (1%)
Median (months)—total	34.2 (32.2–36.1)
Median (months)—p53abn	22.2 (15.4–29.0)
Median (months)—p53wt	34.9 (32.9–36.8)
**Recurrence total**	
Yes	13 (3.6%)
No	353 (96.4%)
**Recurrence p53abn**	
Yes	2 (11.1%)
No	16 (88.9%)
**Recurrence p53wt**	
Yes	11 (3.1%)
No	341 (96.3%)
**DFS p53abn patients**	
Median (months)	21.8 (95% CI 15.4–29.0)
**DFS p53wt patients**	
Median (months)	34.2 (95% CI 32.6–36.6)
**OS p53abn patients**	
Median (months)	21.9 (95% CI 16.4–30.1)
**OS p53wt patients**	
Median (months)	34.2 (95% CI 33.2–37.0)
**Last follow-up status**	
Alive without disease	359 (98.1%)
Alive with disease	3 (0.8%)
Dead of disease	1 (0.3%)
Dead of other cause	3 (0.8%)
**Sites of recurrence**	
Vaginal cuff	6 (46.1%)
Isolated pelvic peritoneal	2 (15.4%)
Pelvic nodes	1 (7.7%)
Para-aortic nodes	1 (7.7%)
Isolated parenchymatous	2 (15.4%)
Extra abdominal	1 (7.7%)
**Therapy at recurrence**	
RT	4 (30.8%)
CHT	1 (7.7%)
Surgery	3 (23.0%)
Surgery + RT	4 (30.8%)
Surgery + RT + CHT	1 (7.7%)
**Response to therapy at recurrence**	
Complete response	9 (69.2%)
Partial response	2 (15.4%)
Stable disease	1 (7.7%)
Progression	1 (7.7%)

RT = radiotherapy; CHT = chemotherapy; DFS = disease-free survival; OS = overall survival.

**Table 5:** Main characteristics of p53abn patients who recurred. PT = patient; PL = pelvic lymphadenectomy; BMI = body mass index; AUB = abnormal uterine bleeding; DFS = disease-free survival; FUP = follow-up; PWC = peritoneal washing cytology; RH = radical hysterectomy; BSO = bilateral salpingo-oophorectomy; PA = para-aortic; G = grading; NED = note evidence of disease; ° = time calculated in months. * The patient underwent surgery at recurrence: histological examination of the lymphadenopathy showed high-grade tumors, p53 showed a mixed expression (mutated and wild-type areas), and PWC was positive. MMR proteins were proficient, and ER and PgR were only scarcely expressed (<1%). She underwent para-aortic radiotherapy. The last FUP was carried out with CT-PET in September 2023.

	Age	History of Cancer	BMI	Symptoms	Type of Surgery	G	Immunohistochemistry	DFS °	Site of Recurrence	FUP Status
Pt 1	69	no	>25	none	PWC, RH, BSO and PL	G2	p-MMR, ER 80%, PgR 80%	11	Vaginal cuff	lost
Pt 2	75	no	>25	AUB	PWC, RH, BSO and PL	G2	p-MMR, ER 15%, PgR 5%	26 *	PA lymph node	NED

## Data Availability

The datasets generated during and/or analyzed during the current study are available from the corresponding authors upon reasonable request.
